# Stressed but Stable: Canopy Loss Decreased Species Synchrony and Metabolic Variability in an Intertidal Hard-Bottom Community

**DOI:** 10.1371/journal.pone.0036541

**Published:** 2012-05-04

**Authors:** Nelson Valdivia, Claire Golléty, Aline Migné, Dominique Davoult, Markus Molis

**Affiliations:** 1 Instituto de Ciencias Marinas y Limnológicas, Facultad de Ciencias, Universidad Austral de Chile, Valdivia, Chile; 2 Centro de Estudios Avanzados de Zonas Áridas (CEAZA), Facultad de Ciencias del Mar, Universidad Católica del Norte, Coquimbo, Chile; 3 Université Pierre et Marie Curie (UPMC), Université Paris 6, Station Biologique de Roscoff, Roscoff, France; 4 Centre National de la Recherche Scientifique (CNRS, UMR 7144), Station Biologique de Roscoff, Roscoff, France; 5 Sediment Ecology Research Group, Scottish Oceans Institute, University of St. Andrews, St. Andrews, United Kingdom; 6 Biologische Anstalt Helgoland, Alfred Wegener Institute for Polar and Marine Research, Section Functional Ecology, Helgoland, Germany; National Institute of Water & Atmospheric Research, New Zealand

## Abstract

The temporal stability of aggregate community properties depends on the dynamics of the component species. Since species growth can compensate for the decline of other species, synchronous species dynamics can maintain stability (i.e. invariability) in aggregate properties such as community abundance and metabolism. In field experiments we tested the separate and interactive effects of two stressors associated with storminess–loss of a canopy-forming species and mechanical disturbances–on species synchrony and community respiration of intertidal hard-bottom communities on Helgoland Island, NE Atlantic. Treatments consisted of regular removal of the canopy-forming seaweed *Fucus serratus* and a mechanical disturbance applied once at the onset of the experiment in March 2006. The level of synchrony in species abundances was assessed from estimates of species percentage cover every three months until September 2007. Experiments at two sites consistently showed that canopy loss significantly reduced species synchrony. Mechanical disturbance had neither separate nor interactive effects on species synchrony. Accordingly, *in situ* measurements of CO_2_-fluxes showed that canopy loss, but not mechanical disturbances, significantly reduced net primary productivity and temporal variation in community respiration during emersion periods. Our results support the idea that compensatory dynamics may stabilise aggregate properties. They further suggest that the ecological consequences of the loss of a single structurally important species may be stronger than those derived from smaller-scale mechanical disturbances in natural ecosystems.

## Introduction

Current rates of species loss have spurred studies testing the influence of diversity on the stability of natural communities [1], [2]. As a result, ecologists recognise today that community stability depends on the temporal dynamics of the species that form the communities [1]. Species abundances can be highly variable over time, influenced by external abiotic factors, internal biotic interactions, and the combination of both [3]. The effect of species' abundance variability on community stability may depend on the degree to which species fluctuations are synchronous or compensatory [4]. Theoretical models indicate that the loss or decline of species can be compensated by others with different environmental tolerances, maintaining relative stability (i.e. invariability) in aggregate, community-level properties like community abundance and productivity [1], [3], [5], [6]. However, field-based evidence that compensatory dynamics maintain community stability is still inconclusive [7], [8], [9], [10].

Community respiration, defined as the sum of metabolic rates across species, is an important aggregate property because of its determinant role in the carbon cycle [11]. In addition, it directly reflects resource availability, representing therefore a powerful indicator of ecosystem “health” and ecological conditions [8]. Several studies show that compensatory species dynamics maintain a steady state in the rates of resource supply and resource use [12], [13], [14], [15]. Therefore, it can be predicted that the degree of synchrony in species abundances may influence the temporal variation in community metabolism [16]. According to Micheli [4], testing this hypothesis needs species attributes to vary independently from those at the community-level. Both, species abundances and community-level metabolism should be measured independently.

In addition, it is still necessary to assess the effect of key species on community stability [17]. On temperate rocky shores, canopy-forming algae are key structural elements that modify the environment and facilitate or suppress the occurrence of other species [18], [19], [20]. Facilitation by canopy-forming seaweeds may occur by alleviating abiotic stress through shading and reduced desiccation [21], [22]. Canopies may prevent recruitment of understorey species by pre-emption or sweeping fronds [23], [24], [25]. In addition, large and dominant species can be more persistent than subordinate ones [26], [27]. So, the presence of a dominant species may increase the stability of aggregate properties [28]. This could be the case of canopy-forming macroalgae, whose structural specialisation of thallus may confer them toughness and resistance to mechanical stress [29]. Moreover, canopy-forming seaweed can be responsible for a significant proportion of community metabolism [30], [31], [32]. These effects on species abundances and community-level properties suggest that canopies influence the temporal variability at both levels of ecological organisation.

**Figure 1:**
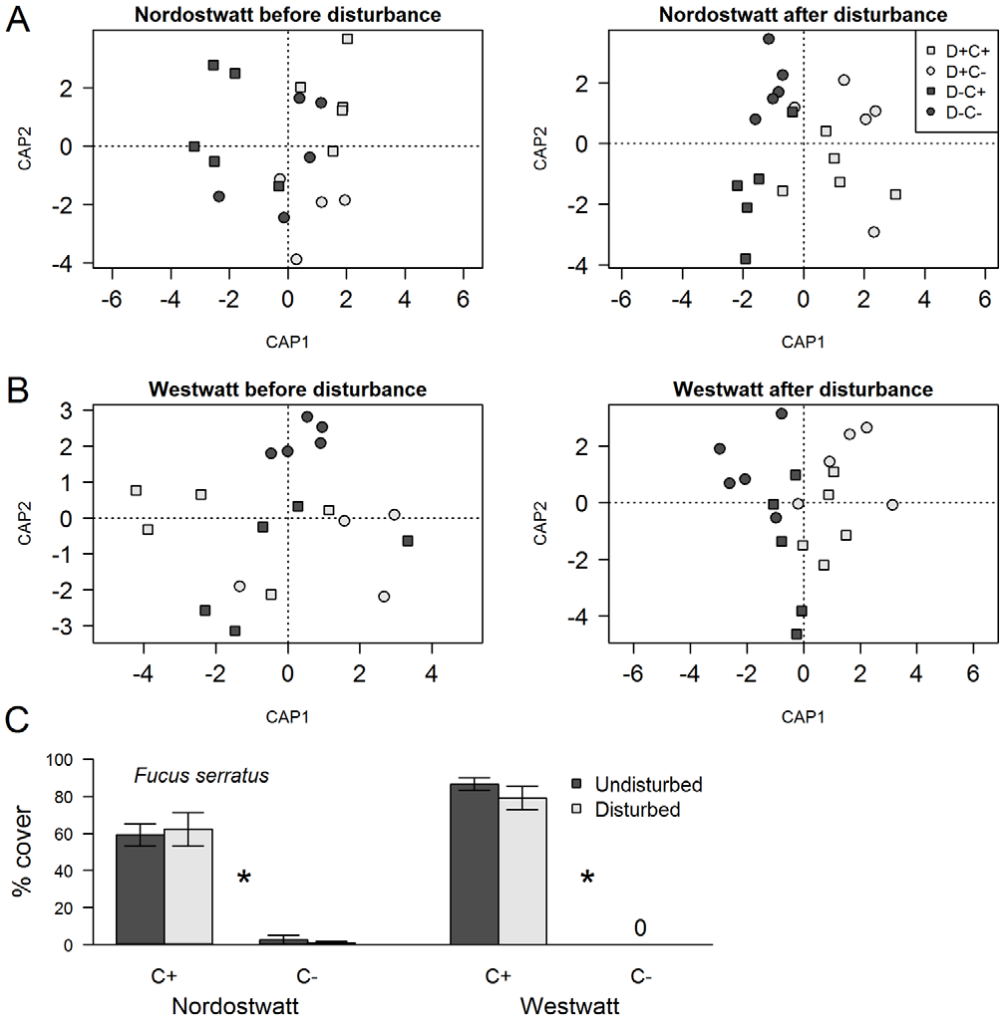
CAP ordination plots of species composition (A) before and (B) 1–3 days after canopy removal and mechanical disturbance treatments, and (C) long-term mean cover of *Fucus serratus* canopy. In panels A and B, the first and second CAP axes explained 41% and 34% of total inertia, respectively, at Nordostwatt and 68 and 17, respectively, at Westwatt. D− and D+ are undisturbed or disturbed treatments, respectively; C+ and C− indicate presence and removal of canopy, respectively. In panel C asterisks indicate significant differences between canopy treatments, and values are given as means ± SEM (n = 5)

Loss of large canopy-forming species is accelerated [33], and mechanical stress on coastal ecosystems increases as a consequence of, for instance, increased storminess [34]. Canopy loss may also exacerbate the effects of additional storm-induced disturbances on community stability. Crashing waves can dislodge or harm benthic organisms on rocky shores [35], and these effects can be disproportionately larger on delicate growth forms than massive canopy-forming seaweeds [29]. Consequently, mechanical disturbances may affect the composition of understorey assemblages without necessarily removing canopies. Since canopies can limit the subset of species able to colonise the substratum [23], the effects of disturbances on the understorey community could be weak when canopies are present, but strong when canopies are removed. Cumulative evidence suggests that canopy removal and mechanical disturbances interactively affect community structure [20], [36], [37]; however, studies linking these interactive effects to stability are still necessary to acquire a mechanistic understanding of the ecological consequences of anthropogenic impacts on natural communities.

Here we tested the separate and interactive effects of canopy removal and mechanical disturbance on species synchrony and the temporal variability in community respiration of intertidal rocky shore communities. Species synchrony was defined as the degree to which species' abundances vary simultaneously over time; for example, species synchrony will be high if most species' abundances show parallel increases or decreases. We measured species abundances and community-level respiration independently, instead of calculating the latter as a scalar function of species attributes [15]. This allowed independent estimators of species- and community-level variability. Factorial field experiments were conducted to test the predictions (1) that canopy removal and mechanical disturbances affect species synchrony and metabolic variability, and (2) that the effects of mechanical disturbance are stronger in communities that have lost the canopy than in communities with a canopy present.

## Materials and Methods

### Study sites

Experiments were conducted in the intertidal at two sites, ‘Westwatt’ (54°11′22″S, 7°52′14″E) and ‘Nordostwatt’ (54°11′8″S, 7°52′26″E), located in the nature reserve of Helgoland Island, German Bight, NE Atlantic. This research adheres to the legal requirements of the Schleswig-Holstein state act of 24 April 1981 (classification number 791-4-37) that declared the rocky shores below the high tide limit in Helgoland a nature reserve and allows ecologists to conduct and maintain manipulative field experiments. The intertidal hard-bottom community at both sites is dominated by the toothed wrack *Fucus serratus*. The most abundant seaweed species of the understorey include crustose coralline algae (mostly *Phymatolithon* spp.) and the turf-forming algae *Cladophora rupestris*, *Chondrus crispus*, and *Corallina officinalis* [38]. During spring and summer, foliose and filamentous ephemeral algae such as *Ulva* spp., *Dumontia contorta*, Ectocarpales, and seasonal Cladophorales are abundant in gaps between the *F. serratus* canopy [39]. The most common sessile invertebrate species are the hydroid *Dynamena pumila*, the polychaete *Spirorbis spirorbis*, and the bryozoan *Electra pilosa*, while conspicuous mobile consumers include the green crab *Carcinus maenas* and several species of periwinkles dominated by *Littorina littorea* [40], [41].

### Experimental design and set-up

Fully factorial experiments were designed with 2 canopy treatments [canopy unchanged (C+) or fully removed (C−)] and 2 levels of mechanical disturbance treatments [undisturbed (D−) or disturbed (D+)] with 5 replicates at each of the 2 sites ( = 40 experimental units).

The experiment was conducted between March 2006 and September 2007 and all manipulations and observations were performed during emersion times. Prior to manipulations, the position of twenty 0.3×0.3 m plots with ≥90% *F. serratus* cover were permanently marked at each site with stainless steel screws. Plot size was adopted from other experiments involving the removal of fucoid canopies [42] and used to minimise the impact of this manipulation on the natural *F. serratus* population. Plots were positioned on flat, gently sloping surfaces lacking larger crevices in order to ensure homogeneity among plots and correct fitting of benthic CO_2_ chambers (see *Community metabolism* section below). To reduce the possibility of effects from adjacent canopies, all *F. serratus* plants were removed from a 50 cm wide band around C− plots so that adjacent *F. serratus* specimens could not reach into these plots.

At the start of the experiment in March 2006, all *F. serratus* specimens were completely removed with a knife from 10 plots at each site (C−), while canopies of remaining 10 plots were left untouched (C+). This treatment was repeated every three months until the end of the study. Care was taken that remaining organisms were not damaged. Five plots were randomly selected from each canopy treatment to apply the mechanical disturbance treatment (D+) once at the start of the experiment in early spring. We selected this timing of disturbance in order to match the main settlement period of several intertidal algae in Helgoland [43], [44]. Mechanical disturbance treatment consisted of a biomass removal with 50% of the effort required to remove all organisms (except crustose algae) from a plot. In pilot tests, 36 scratches of a 2 cm wide chisel were needed to remove all organisms from a 0.3×0.3 m area. Thus, 18 chisel scratches were haphazardly applied to selected plots at each site. In the D+C+ treatment combination, some *F. serratus* specimens were damaged or dislodged due to the mechanical disturbance. Nevertheless, this effect can be seen as analogous to that of any mechanical disturbance naturally impacting these assemblages (e.g. wave action). Therefore, we assumed that mechanical dislodgement of *F. serratus* in D+C+ plots did not prevent logic interpretation of separate and interactive effects of canopy removal and mechanical disturbances on stability [37].

### Sampling

#### Species synchrony

Percentage cover of each macro-epibenthic species (>5 cm) was estimated per plot to the nearest 1% by the same observer (i) prior to, (ii) 1–3 days after, and (iii) every three months after the initial treatment in March 2006 until September 2007. Percentage cover directly reflects resource availability in marine hard-bottom communities, where competition for settlement surfaces is regarded as a pivotal driver of species dynamics [45]. In addition, percentage cover is routinely used as a proxy for species abundances [46], and can be significantly correlated with biomass [47]. Because the abundance of organisms attached to primary and secondary substrate was considered and due to the multi-layered structure of communities, cover estimates for the sum of all species on each plot (i.e. total community cover) were not limited to 100%. Accordingly, it was assumed that percentage cover was appropriate to assess temporal dynamics in species abundances. In order to corroborate the correlation between percentage cover and biomass, we determined at the end of the experiment dry mass (hereafter biomass) of the assemblage of each plot in Nordostwatt after all species cover was removed and dried to constant weight at 60°C.

**Figure 2:**
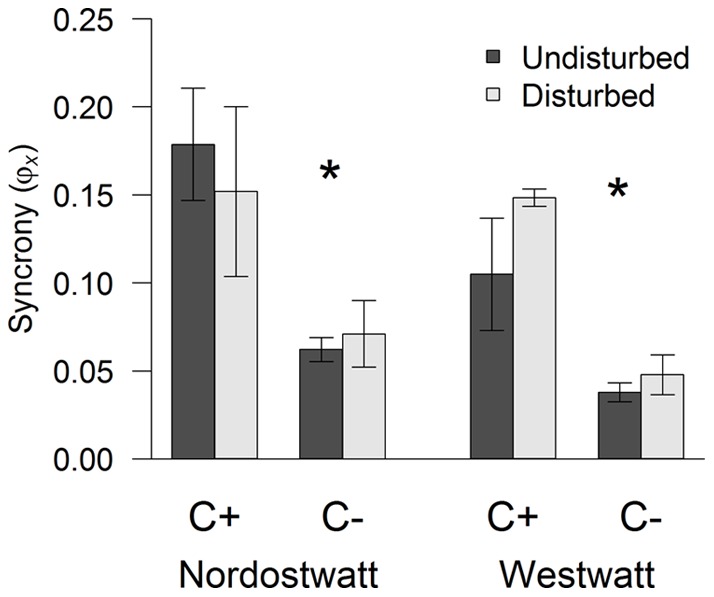
Effects of removal of *Fucus serratus* canopy and mechanical disturbance on synchrony in species abundances (*ϕ_x_*) at Nordostwatt and Westwatt. Values of *ϕ_x_* = 1 indicate perfect synchrony; *ϕ_x_* = 0 indicates perfect asynchrony. D– and D+ indicate undisturbed and mechanically disturbed treatments, respectively. Values are given as means ± SEM (n = 5). Asterisks denote significant differences between canopy treatments.

Taxa were identified to the lowest possible taxonomic level in the field, usually to species level. When appropriate, specimens of unidentified taxa were taken from adjacent areas to the laboratory for species identification. Four taxa could only be identified to genus level (*Porphyra* spp., *Phymatolithon* spp., *Sagartia* spp., and *Ulva* spp.), while small spionids were grouped at the family level (Spionidae) and small brown filamentous algae at the order level (Ectocarpales).

We used species abundance data obtained before treatments to confirm that the experimental communities were similar in terms of species composition. Species abundances estimated within 3 days after manipulations were used to validate treatment efficacy (see statistical analyses, below). Three-monthly estimates on species abundances were used to test our hypotheses as explained on the following lines.

We used the *ϕ_x_* statistic to measure community-wide synchrony [48] as:
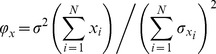
(1)where *ϕ_x_* describes the sum of the standard deviations of all *N* individual species (*x_i_*) with respect to the variance of the summed total and is standardised between 0 (perfect asynchrony) and 1 (perfect synchrony; i.e. most species' abundances show positively correlated changes over time). The *ϕ_x_* statistic uses the fact that the variance of an aggregate property can be partitioned into the sum of all species variances plus the sum of all pair-wise species covariances [49,50] such that:
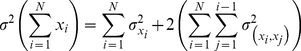
(2)The *ϕ*x statistic does not make any assumption on the magnitude and distribution of species abundances and variances, allowing quantitative comparisons of communities with different species richness [48]. The percentage cover of F. serratus was included in the calculations of species synchrony. The analysis was, however, repeated after excluding F. serratus abundance from the dataset in order to check for consistency of results (see results).

#### Community metabolism

CO_2_-fluxes due to respiration at the rock-air interface were measured at low tide 6, 16, and 18 months after starting the experiment. All metabolic measurements were conducted during daytime. Due to logistic constrains, community metabolism was measured at only one site (Nordostwatt). We used a benthic chamber made of a transparent Perspex dome with a 0.3×0.3 m transparent Perspex base, equalling a total volume of 18.9 L [51]. Each plot was completely enclosed by the chamber base and air-tightly sealed with neutral silicon. Changes in CO_2_ mol fraction were measured by an infrared CO_2_ gas analyser (LI- 800; LI-COR Inc., Lincoln, NE, USA) and recorded with a data logger (LI-1400; LI-COR Inc.) every 15 seconds. Each plot was incubated for ca. 20 min and CO_2_ fluxes then calculated from the absolute value of the slope of CO_2_ concentration (μmol_CO2_ mol_air_^−1^) against time. Results were expressed as carbon units (mmol C m^−2^ h^−1^), assuming a molar volume of 22.4 L mol^−1^ at standard temperature and pressure. Measurements were carried out in darkness, by covering the chamber with an opaque polyethylene sheet, to assess community respiration (CR). The coefficient of variation of CR (*V_CR_*  =  sd/mean_CR_) was then calculated from the three measurements made during the experiment of each plot. Coefficients of CR variation were used to test the hypothesis of separate and interactive effects of canopy removal and mechanical disturbance on the temporal variation in community metabolism.

In addition, 6 months after the onset of the experiment we measured CO_2_-fluxes under light conditions to assess net primary productivity (NPP). During the incubations, air temperatures in the chamber ranged between 19 and 25°C (July), and between 9 and 16°C (October). Nevertheless, air temperatures varied over time during the incubations by −0.45% in July and +0.71% in October.

**Table 1:** ANOVA of the effects of canopy removal and mechanical disturbance on synchrony in species abundances, temporal coefficient of variation of community respiration (*V_CR_*), and understorey community respiration.

Source of variation										
	Species synchrony^1^					Understorey species synchrony^1^				
	DF	MS	F	P	MS_den_.	DF	MS	F	P	MS_den_.
Site = S	1	0.0225	3.33	0.077	Residuals	1	0.0060	2.6364	0.114	Residuals
Canopy = C	1	0.2053	31.14	**<0.001**	Pooled	1	0.0428	19.5975	**<0.001**	Pooled
Disturbance = D	1	0.0017	0.25	0.621	S × D	1	0.0027	1.1685	0.288	Pooled
S × C	1	0.0001	0.01	0.921	Pooled	1	0.0004	0.1684	0.684	Residuals
S × D	1	0.0114	1.7	0.202	Residuals	1	0.0015	0.6358	0.431	Residuals
C × D	1	<0.0001	<0.1	0.977	Pooled	1	0.0005	0.2166	0.645	Pooled
S × C × D	1	0.008	1.19	0.284	Residuals	1	0.0013	0.5604	0.459	Residuals
Residuals	32	0.0068				32	0.0023			
Pooled	34	0.0066				36	0.0022			
Transformation	Square-root					Square-root				

	Temporal variation in community respiration (*V_CR_*)^2^					Understorey community respiration^2^				
	DF	MS	pseudo-F	P _Monte Carlo_		DF	MS	pseudo-F	P _Monte Carlo_	
Canopy = C	1	0.8469	18.22	**0.017**		1	0.1027	0.04	0.859	
Disturbance = D	1	0.1159	2.49	0.193		1	1.9911	0.72	0.402	
C × D	1	0.009	0.19	0.687		1	0.0046	0.00	0.959	
Residuals	4	0.0465				8	2.7546			
Transformation	None					None				

The abundance of *F. serratus* was included in the analyses of synchrony (“Species synchrony”). The analysis was repeated after excluding *F. serratus* abundance in order to check for consistency of results (“Understorey species synchrony”). DF, SS, and MS stand for degrees of freedom, sum of squares, and mean squares, respectively. Significant P-values are in bold.

1 For species synchrony, terms used as denominator for F-ratio are listed in the column *MS_den_.* Error terms with highly conservative P-values (>0.25) were removed from the model. Synchrony was measured at Nordostwatt and Westwatt (n = 40). ^2^For *V_CR_* and understorey community respiration (n = 2 and 3, respectively), the distribution of the F-ratio was obtained after 999 Monte Carlo samples of raw data. *V_CR_* and understorey community respiration were measured at Nordostwatt.

Due to the dominance of the canopy, it is likely that canopy itself could explain any observed differences in metabolism between C+ and C− plots. In order to compare only understorey species, we obtained an additional sample of CR at the end of the experiment after having removed the canopy from the C+ plots (i.e. understorey community respiration). These understorey CR values were obtained immediately after the last CR measurements.

### Statistical analysis

Permutational multivariate analysis of variance (PERMANOVA [52]) were used to test (i) whether experimental communities allocated to different treatment combinations varied in their species composition before manipulations, (ii) whether canopy removal affected species composition of understorey communities, and (iii) to validate the efficacy of the mechanical disturbance treatment. These analyses were complemented with a canonical analysis on principal coordinates (CAP [53]), a constrained multivariate method that uses an *a priori* hypothesis to produce an ordination plot, allowing us to detect patterns that could be masked by overall dispersion in unconstrained methods such as multidimensional scaling. CAP plots were based on a matrix of factors (canopy and mechanical disturbance) fitted to a matrix of Bray-Curtis dissimilarities calculated from presence/absence-transformed data. PERMANOVA and CAP analyses were conducted for each site, before, and 1–3 days after the application of treatments.

**Figure 3:**
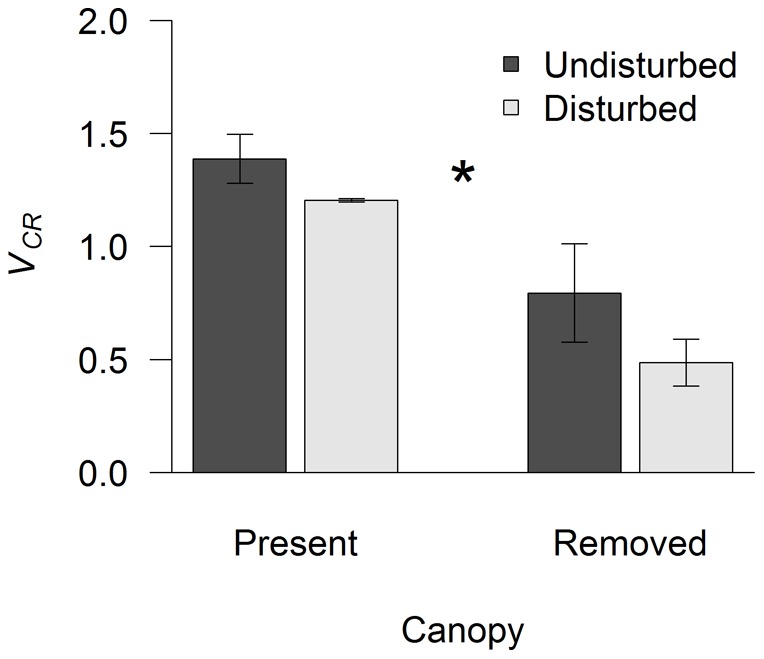
Effects of canopy removal and mechanical disturbance on the coefficient of temporal variation of community respiration (*V_CR_*) at Nordostwatt. D− and D+ indicate undisturbed or mechanically disturbed treatments, respectively. Values are mean (± SEM, n = 2). Asterisk denotes a significant difference between canopy treatments.

**Figure 4:**
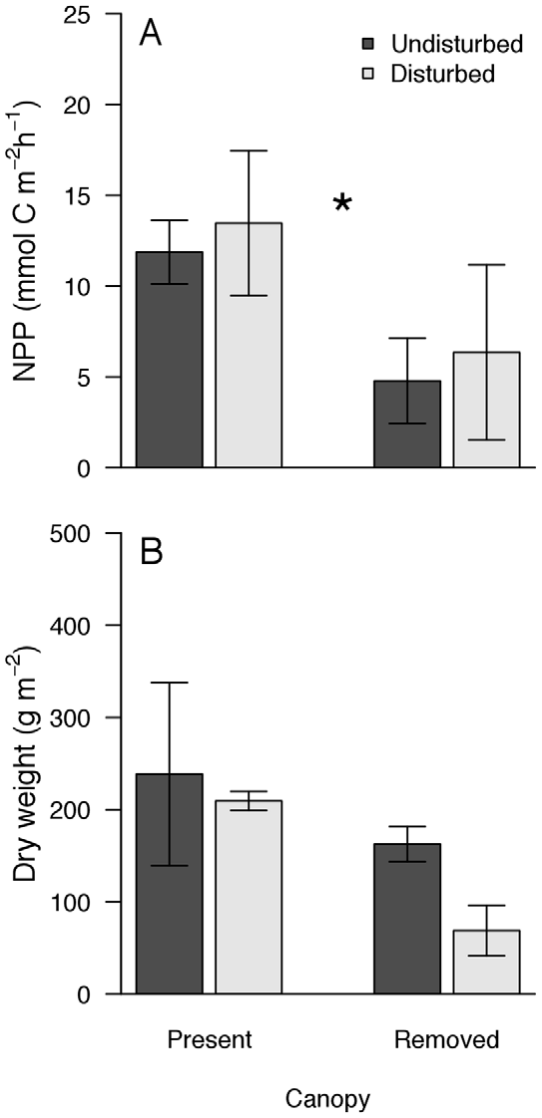
Effects of canopy removal and mechanical disturbance on the Net Primary Productivity (NNP, panel A) and biomass (B) at Nordostwatt. D− and D+ indicate undisturbed or mechanically disturbed treatments, respectively. NPP and biomass were obtained 6 and 18 months after the onset of the experiment, respectively. Values are mean (± SEM, n = 5). Asterisk denotes a significant difference between canopy treatments.

Treatment effects on species synchrony were tested using a 3-way mixed ANOVA with canopy (2 levels: C+ or C−) and mechanical disturbance (2 levels: D− or D+) as fixed factors, and site (2 levels: Nordostwatt and Westwatt) as a random factor. Highly conservative (P>0.25) error terms were removed from the model and F-ratio statistics were then calculated with a pooled denominator [54]. Net primary productivity and biomass were analysed with separate 2-way ANOVAs. Homogeneity of variance was graphically explored by means of residuals-vs.-fits and normal Q-Q plots. Data of species synchrony were square root transformed to achieve homoscedasticity.

**Table 2:** Relative contribution (%) of species to Bray-Curtis dissimilarities between assemblages with and without *Fucus serratus* canopy. “**↑**”, “**↓**”, and “**-**” denote positive, negative, and no effect of the loss of canopy on species abundance, respectively.

	Months after the start of the experiment											
Species	3		6		9		12		15		18	
**Nordostwatt**												
*Cladophora rupestris*	9.6	**↓**	**13.8**	**↓**	**17.2**	**↓**	10.1	**↓**	11.6	**↓**	**12.6**	**↓**
*Cladophora sericea*	**16.9**	**↑**	0.0	**-**	0.0	**-**	0.0	**-**	**14.5**	**↑**	1.7	**↑**
*Ulva* spp.	7.5	**↑**	11.7	**↑**	6.6	**↑**	**13.0**	**↑**	6.4	**↓**	11.2	**↑**
*Chondrus crispus*	6.0	**↑**	9.1	**↑**	9.1	**↑**	6.3	**↑**	6.4	**↑**	6.9	**↑**
*Dumontia contorta*	2.1	**↑**	0.0	**-**	0.0	**-**	8.8	**↑**	7.2	**↑**	0.8	**↓**
*Ceramium virgatum*	1.3	**↑**	1.3	**↑**	2.4	**↑**	2.1	**↑**	4.4	**↑**	9.7	**↑**
*Corallina officinalis*	4.0	**↓**	7.8	**↑**	11.2	**↓**	7.3	**-**	6.9	**↓**	10.3	**↓**
*Phymatolithon* spp.	8.8	**↓**	6.4	**↓**	1.6	**↑**	5.0	**↓**	11.2	**↓**	10.6	**↑**
*Hildenbrandia rubra*	4.5	**↓**	4.7	**↓**	8.8	**↑**	6.2	**↑**	2.5	**↓**	4.8	**↑**
*Spirorbis spirorbis*	8.5	**↓**	7.1	**↓**	4.5	**↓**	3.3	**↓**	0.5	**↓**	2.5	**↓**
**Westwatt**												
*Cladophora rupestris*	6.5	**↓**	8.7	**↓**	10.0	**↓**	5.9	**↓**	6.6	**↓**	10.4	**↓**
*Cladophora sericea*	7.2	**↑**	0.6	**↑**	2.7	**↑**	2.3	**↑**	5.6	**↑**	0.0	**-**
*Ulva* spp.	**17.5**	**↑**	**22.1**	**↑**	4.9	**↑**	**12.7**	**↑**	9.8	**↑**	11.4	**↑**
*Chondrus crispus*	7.1	**↑**	7.2	**↑**	**13.1**	**↑**	5.2	**↑**	8.2	**↑**	8.0	**↑**
*Mastocarpus stellatus*	6.4	**↑**	8.0	**↑**	10.3	**↓**	7.6	**↓**	**13.8**	**↑**	**14.2**	**↑**
*Corallina officinalis*	5.5	**↓**	7.1	**↑**	12.5	**↓**	7.9	**↓**	9.3	**↑**	12.2	**↓**
*Phymatolithon* spp.	13.3	**↓**	6.4	**↓**	2.2	**↓**	8.5	**↓**	6.7	**↓**	12.2	**↓**
*Hildenbrandia rubra*	5.6	**↓**	8.5	**↓**	9.3	**↓**	5.7	**↓**	6.2	**↓**	5.8	**↓**

Highest contributions for each site and sample date are in bold.

Due to harsh weather conditions, we were only able to consistently quantify community respiration over time on 2 plots for each treatment combination. At the last sample date (18 months), we were able to measure understorey community respiration on 3 replicate plots. Distributional assumptions cannot be assessed in a design with very low replication. Therefore, we obtained distribution free F-statistics for main and interactive treatment effects on *V_CR_* and understorey community respiration after 999 Monte Carlo samples of raw data; the P-value was calculated as the percentile rank of the observed F-ratio among the samples of F-values [55], [56]. This method does not rely on homogenous variances [56], which makes it preferable over rank-based tests (e.g. Scheirer-Ray-Hare test [57]), for which the assumption of equality of variances across groups still applies [54].

Multivariate analyses were conducted in order to identify the species contributing most to differences in community structure between treatments. Separately for each sample date and site, we conducted similarity percentage routines (SIMPER), which consisted in calculating Bray-Curtis dissimilarities between all pairs of treatments and then breaking down the average between-group dissimilarity into the contribution from each species [58].

SIMPER routines were performed using PRIMER v.5. All other statistical analyses were conducted using the R environment version 2.13.1 [59].

## Results

We identified a total of 51 species in the understorey assemblages at both sites. Invertebrates were represented by 27 species, red algae by 15, brown algae by 5, and green algae by 4 (see reference [60] for a complete species list). A total of 48 and 47 species were identified in Nordostwatt and Westwatt, respectively. Apart from crustose coralline algae like *Phymatolithon* spp. dominating understorey assemblages at both sites, Cladophorales like *C. rupestris* (mean cover ± standard error of the mean: 20.3±1.8%) and *Cladophora sericea* (6.9±0.2%) were more abundant at Nordostwatt, and turf-forming algae like *C. crispus* (21.2 ±1.7%) and *Mastocarpus stellatus* (7.2±1%) were more abundant at Westwatt. Biomass of assemblages measured at the end of the experiment was linearly and significantly correlated to total percentage cover (biomass [g m^−2^] = –17.9+0.6 total cover [%], *r^2^* = 0.695, P<0.001).

Prior to the manipulations, community structure was similar across treatments (Fig 1A and 1B, P>0.4 for canopy, disturbance, and interaction for both sites). PERMANOVA indicated that community composition following initial treatments was significantly affected by the mechanical disturbance only at Nordostwatt (pseudo-F_1, 16_ = 3.35, P = 0.005). Nevertheless, CAP ordination plots showed comparable levels of differentiation between disturbed and undisturbed plots following initial treatments at both sites (Fig 1A and 1B), indicating the efficacy of this treatment in affecting community composition. During the experiment and averaged across the 7 sample dates, the cover of the *F. serratus* canopy was ca. 60–90% in the C+ plots, and remained below 10% in the C− plots (Fig 1C). These differences between canopy treatments were statistically significant at both sites (ANOVA, P<0.01 for both sites), and no separate or interactive effects of mechanical disturbances on *F. serratus* cover were found (P>0.3 for both sites).

Species synchrony indicates the extent to which species temporal changes are parallel and was, on average, a significant 60% lower in C− than C+ communities (Fig 2, Table 1). Mechanical disturbance showed no main effect on species synchrony, and the effects of canopy removal were independent of mechanical disturbance as indicated by a missing canopy × disturbance interaction (Table 1). No significant differences between sites were found. Effects on species synchrony remained unchanged after excluding canopy abundance from the analysis (Table 1).

Canopy removal significantly reduced temporal variation in community respiration by, on average, 50% and this effect was independent of mechanical disturbance treatments (Fig 3, Table 1). Mechanical disturbance did not affect temporal variation of community respiration. The temporal mean of community respiration was 20.3 and 3 mmol C m^−2^ h^−1^, for C+ and C– treatments, respectively, indicating that canopy removal reduced community respiration by about one order of magnitude. Neither the separate nor the combined effects of canopy removal and mechanical disturbance affected understorey ecosystem respiration (Table 1).

After 6 months of experimentation, net primary productivity significantly varied only between C+ and C– treatments (Fig 4, P = 0.002, P = 0.92, P = 0.79 for canopy removal, mechanical disturbance, and interaction, respectively). At the end of the experiment, biomass tended to respond negatively to the interaction between canopy removal and mechanical disturbance (Fig 4). ANOVA, however, showed non-significant effects of treatments on biomass (P>0.1 for canopy, disturbance, and interaction).

SIMPER routines conducted on data pooled across mechanical disturbance treatments showed that up to 10 and 8 species in Nordostwatt and Westwatt, respectively, explained>60% of dissimilarity between canopy treatments (Table 2). In general, removal of canopy had a strong negative effect on the abundance of *C. rupestris*, *Phymatolithon* spp., and the polychaete *Spirorbis spirorbis* (Table 2). On the other hand, abundances of *C. crispus* and *M. stellatus,* in addition to filamentous algae such as *C. sericea*, *Dumontia contorta*, and *Ceramium virgatum*, and the foliose green algae *Ulva* spp. increased in response to canopy removal (Table 2).

## Discussion

Our results showed that species synchrony significantly decreased as a result of the loss of canopy, indicating that compensatory dynamics may have been strengthened upon canopy removal. Canopy removal led to a decrease in net primary productivity (NPP) and temporal variation of community respiration (*V_CR_*). Neither separate nor interactive effects of mechanical disturbance on species synchrony, NPP, and *V_CR_* were observed. These results support the notion that compensatory species dynamics stabilise aggregate community properties. They also indicate that canopy removal, but not smaller-scale mechanical disturbances, may significantly affect mechanisms contributing to community stability.

Canopy-forming species usually reduce the variability in environmental factors [21,22]. Since seasonal changes in, for example, temperature and light, can be large in Helgoland [44], experimental canopy removal exposed the understorey assemblage to a wide range of environmental conditions. Different species may be competitively dominant at different times [61], which can foster the occurrence of asynchronous, compensatory dynamics among species exposed to large environmental variability [3,62]. In our experiment, for instance, canopy loss had lead to an average increase in light regime of ca. 400% during summer months [63]. Such changes may have detrimental effects for some understorey species, like low-light adapted seaweeds, but may have beneficial effects for high-light adapted macroalgae. In fact, abundance of low-light adapted crustose red algae (e.g. *Phymatolithon* spp.) declined in plots where the *F. serratus* canopy was removed, while abundance of high-light adapted green algae (e.g. *Ulva* spp.) increased here relative to control plots. Moreover, insulation effects of *F. serratus* canopies at the study site during low tide may reduce air temperature in the understory for several hours during sunny afternoons by up to 20°C (M. Molis, unpublished data). Changes in sedimentation rate, light, and temperature regimes as a consequence of canopy loss have been reported as important abiotic drivers of community structure during subsequent succession in seaweed dominated communities [64,65]. Therefore, differential abilities of understorey species to cope with enhanced environmental variability upon canopy loss probably decreased species synchrony.

According to theoretical models, decreased species synchrony should lead to increased stability of aggregate properties [3,6]. Our results support this prediction, as *V_CR_* significantly decreased upon canopy removal. Asynchronous dynamics, due to contrasting environmental responses of understorey species, may have maintained the steady state in the rates of resource supply and resource use. Differences in life history traits between low-light and high-light adapted species, for instance, may have led to asynchronous variation in metabolic activity [66]. Therefore, it is likely that compensatory dynamics in metabolic functions may well occur, at least, between these two functional groups. This hypothesis however still needs to be tested through proper experimentation.

On the other hand, the temporal variability in the abundance or metabolism of the dominant species may also affect the community-level stability. Canopy-forming seaweeds can significantly contribute to the metabolism of benthic habitats [30,31,32]. Accordingly, the significant negative effects of canopy removal on NPP and CR, and the fact that understorey community respiration did not differ between C+ and C– treatments indicates that *F. serratus* canopy comprised the bulk of productivity and respiration of un-manipulated communities. Thus, the steady state between resource supply and resource use of the community probably depended mostly on the metabolic activity of *F. serratus*.

These results contradict previous work showing that increased dominance by one or few species increases the stability of the total community [26,27,28]. Dominance can enhance community stability when the dominant species are more resistant to events of destruction than subordinate species [26,67]. On the contrary, the small attachment area of *F. serratus* holdfasts may result in dislodgement by dragging forces and cobble bash. Single storm events, for instance, may remove across the entire study site up to 60 % of *F. serratus* canopy (I. Bartsch, unpublished data). Winter losses of *F. serratus* cover could be positively correlated with significant wave height, as shown for giant kelps in California [68,69] and a dominant fucoid in southern New Zealand [70]. In addition, recent manipulative work shows that strengthened compensatory dynamics upon canopy loss can stabilise the total community abundance [71]. Therefore, dominance and monopolisation of resources would actually reduce stability if the dominant species were prone to large variations through time.

In our study, mechanical disturbance had no significant effect on species synchrony, community productivity, and stability, although disturbance can strongly affect species diversity and composition in different benthic systems [72,73]. High seasonality in recruitment patterns and quick re-colonisation may explain the lack of separate and interactive effects of mechanical disturbances. We applied a pulse event of destruction at the onset of the experiment in March, before the main settlement period of several ephemeral, corticated, and leathery algae [44], and during the main reproductive period of *F. serratus* and *F. vesiculosus* [43]. This indicates that propagules of these species could have been available when mechanical disturbances were applied. Covers higher than 20% of *F. serratus* recruits, however, were observed between June and December, and no recruit of *F. vesiculosus* was observed on our plots during the experiment (N. Valdivia, unpublished data). Patches of empty substratum can be re-colonised by both local propagule dispersal and lateral expansion of species with clonal or colonial growth [74,75]. Rapid re-colonisation by expansion of adults could have prevented disturbance-generated patches of habitats from being available during the main settlement period of algae. Variability during early stages of colonisation, in addition to priority effects of the species that happen to colonise first, can determine much of the subsequent dynamics in the assemblage [76,77]. Extreme storms, nevertheless, can be observed every ca. 2 years in the Atlantic basin [78]. At the temporal scale of our study, therefore, timing and frequency of mechanical disturbance seemed to be appropriate to mimic the impact of extreme winter storms.

Some caution should be used to interpret our results. We assumed that abundance and energy use are equivalent measures of community function [15]. The validity of this assumption depends, however, on unmeasured variables such as body size distribution [15,79,80]. On the other hand, calculation of variation in community respiration was constrained by weather conditions and tides to only 3 repeated measurements, indicating the need for longer-term datasets of *in situ* metabolic community functions. Finally, lack of interactive effects between stressors observed in this study agrees with previous manipulative field-based experiments [64,81], but it contradicts a major synthesis of ca. 170 laboratory-based studies [82]. These opposing results hint at the need for more manipulative and field-based experiments accounting for interactive effects of simultaneous stressors on ecosystems [37].

Through manipulative experiments and assessing eco-physiological variables such as species percentage cover, biomass, NPP, and CR, we have tested in natural conditions the effects of multiple anthropogenic stressors on mechanisms that maintain community stability. Our analyses showed that stressors related with storminess may affect species and community-level variability by removal of dominant canopies, but not necessarily by associated smaller-scale mechanical disturbances. These results shed light on the mechanisms that may drive the response of communities facing present and future anthropogenic pressures.
